# Supplementary figures and images for: Malaria parasites can optimize transmission investment by sensing two within-host cues
Source: PLoS Biol. 2025 Oct 9;23(10):e3003081. doi: 10.1371/journal.pbio.3003081 (PMC12551962; doi:10.1371/journal.pbio.3003081)

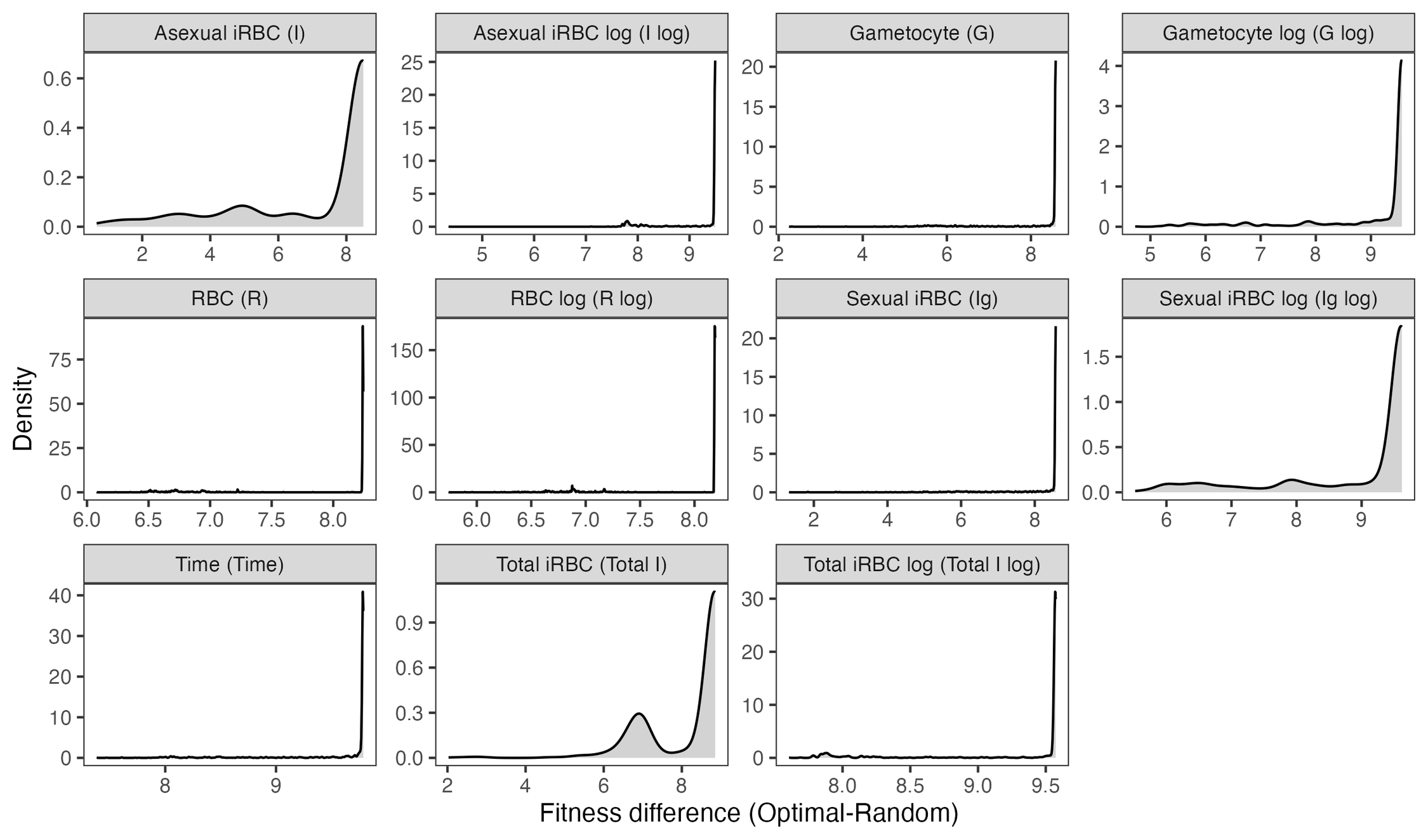

Supplement: S1 Fig — We conducted 1000 infection simulations for parasites sensing different cues, with each simulation using randomly generated transmission investment strategies. The fitness values of these simulations were compared to the fitness of parasites adopting the optimal transmission investment strategy. Each histogram represents the difference between the optimized fitness and the fitness obtained from the randomly generated strategy. The fitness of the random strategies is always lower.The data and code needed to reproduce S1 Fig can be found in DOI: 10.5281/zenodo.17114049, plos-bio_R-notebook.Rmd file (l296-318) and S6 Data. (TIFF) [file pbio.3003081.s001.tif]

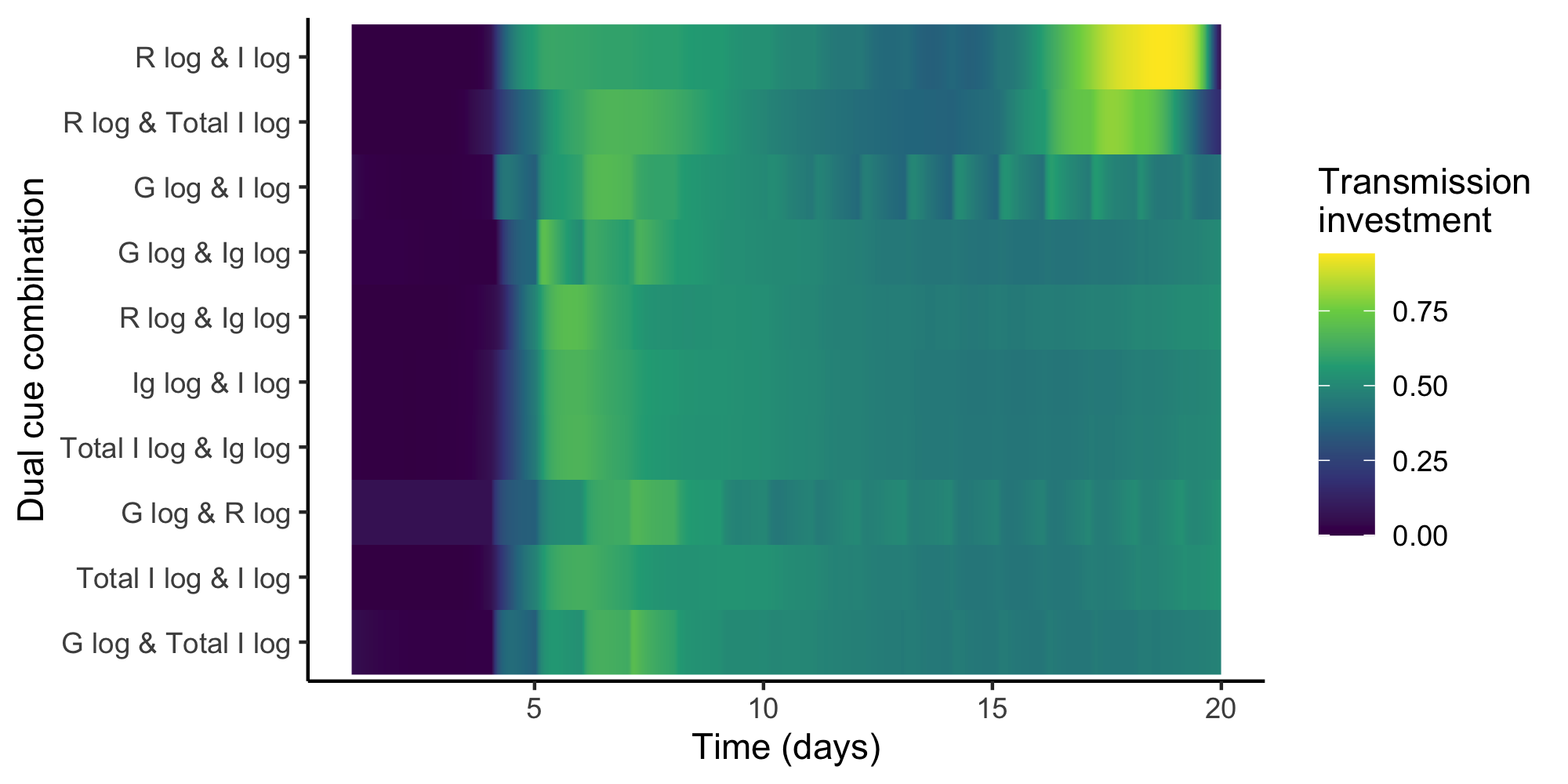

Supplement: S2 Fig — Transmission investment is plotted for all possible dual cue combinations, with heatmaps ranked in descending order (from top to bottom) based on their associated cumulative transmission potential. The data needed to reproduce S2 Fig can be found in S7 Data. (TIFF) [file pbio.3003081.s002.tif]

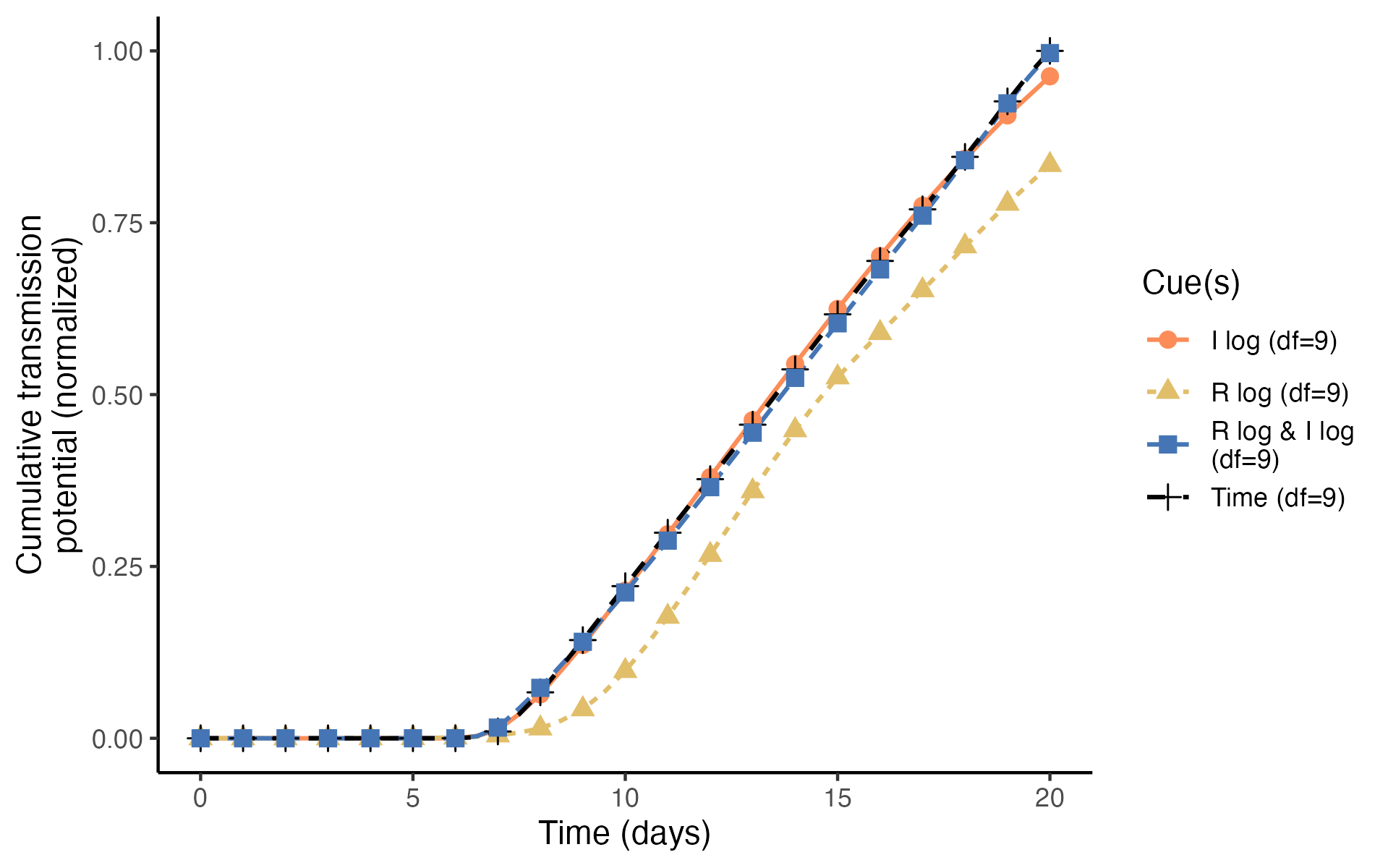

Supplement: S3 Fig — The cumulative transmission potential of parasites sensing time, the best performing dual cue combination (R log & I log), or the individual single cues (I log, R log) are indicated with a black cross, blue square, orange circle, and yellow triangle, respectively. The cumulative transmission potential is normalized with respect to the fitness of the best time-varying strategy. Note that time, the dual cue combination, and I log all permit an initial delay in transmission investment, while R log does not; the fitness advantage of this delayed strategy can be clearly viewed on these plots as fitness accrues faster. Only time and the dual cue combination lead to terminal investment, leading to additional fitness gains from day 18 onward. The data needed to reproduce S3 Fig can be found in S8 Data. (TIFF) [file pbio.3003081.s003.tif]

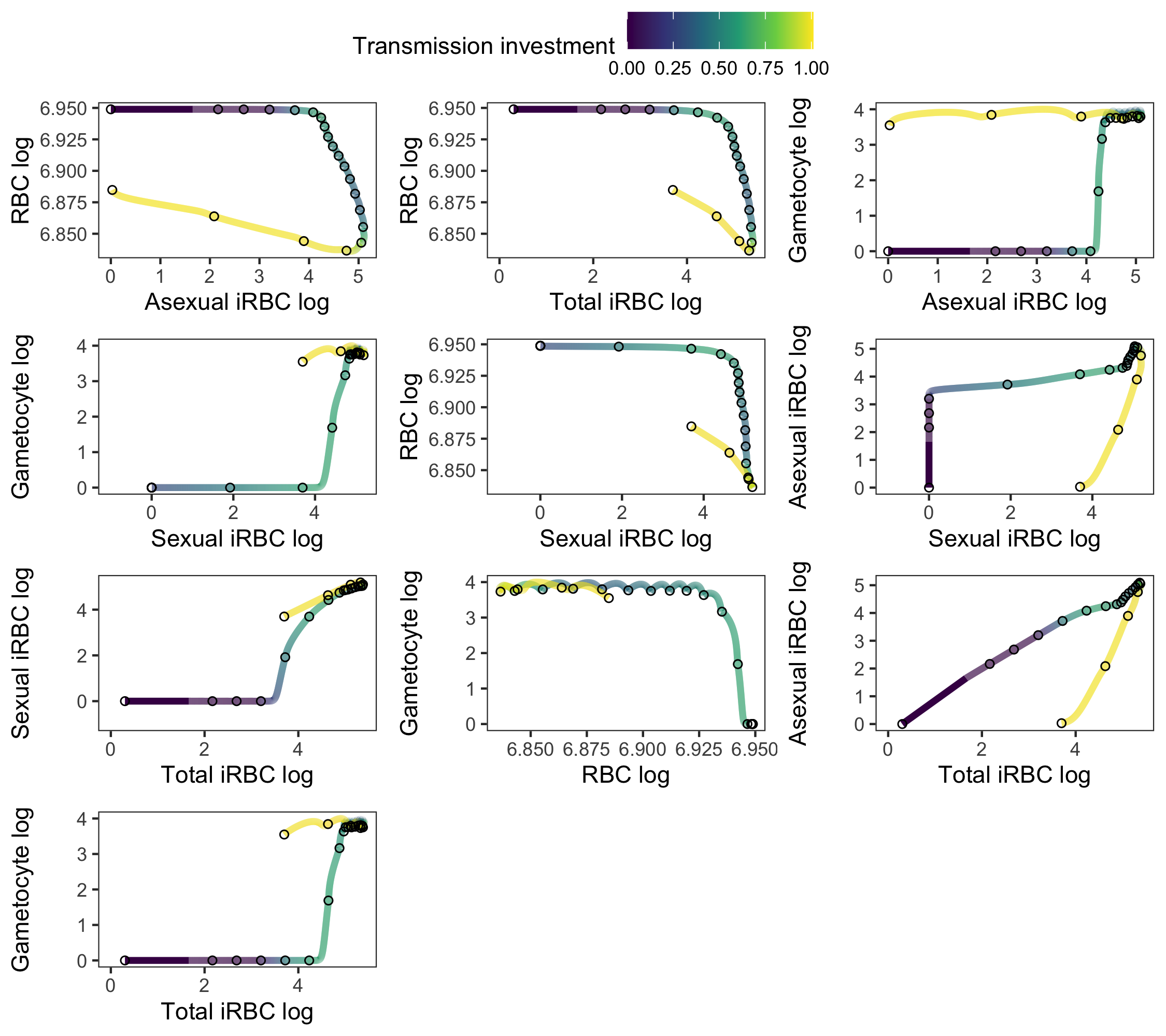

Supplement: S4 Fig — Graphs display the two-dimensional reaction norm and infection trajectories for parasite sensing time with nine degrees of freedom. The trajectories depict the dynamics of the cues (indicated on the x- and y-axes) along with the corresponding transmission investment (represented by the colour of the line). Note that the dynamics of gametocytes and sexual iRBC are hard to display completely given that these densities start off at zero. The distance between each hollow circle represents one day. The data needed to reproduce S4 Fig can be found in S3 Data. (TIFF) [file pbio.3003081.s004.tif]

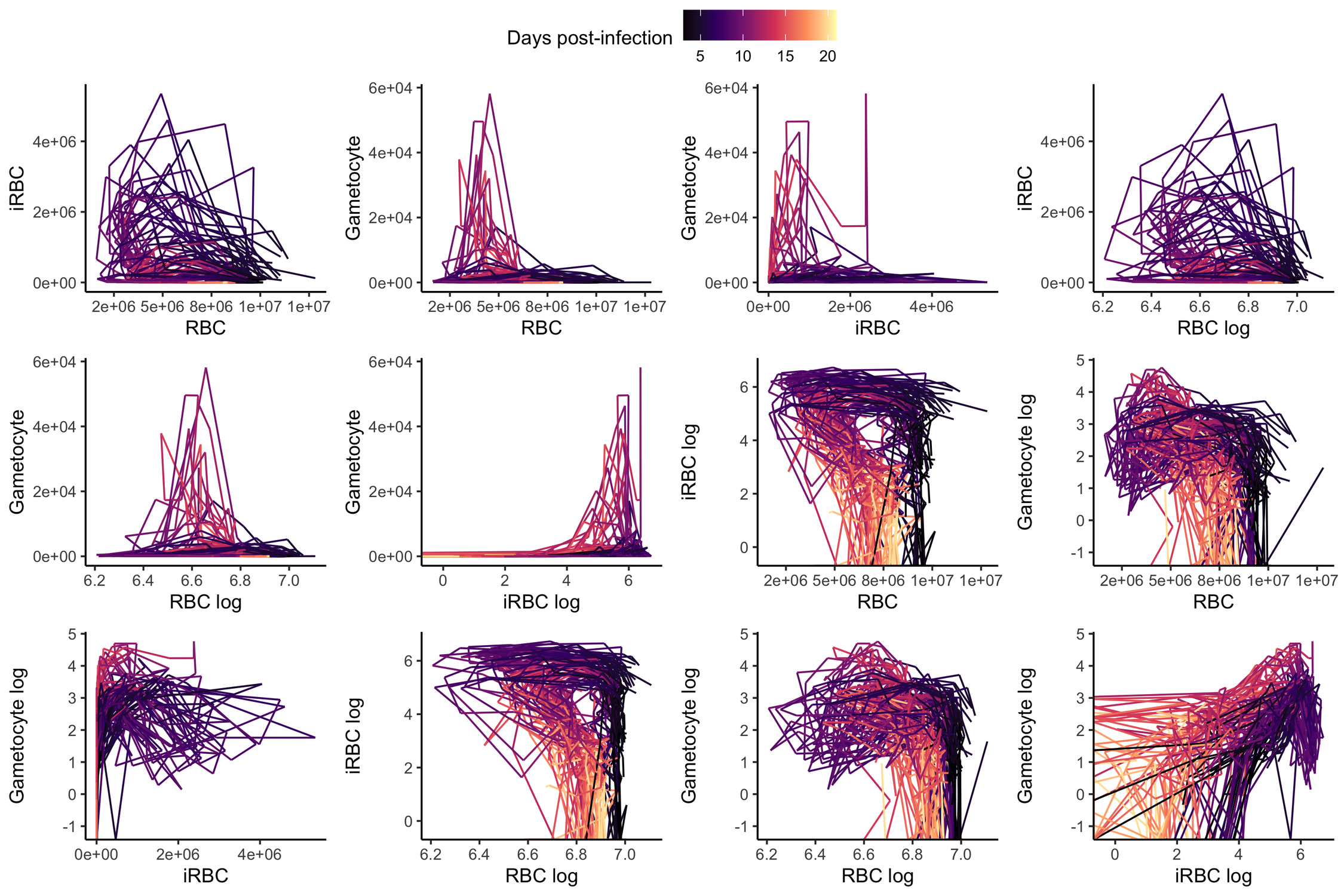

Supplement: S5 Fig — To identify combinations of within-host variables that exhibit a looping relationship, we plotted all possible permutations of experimentally derived P. chabaudi variables. Each line represents the dynamics of a single strain of infecting parasite. The line colour indicates the progression of time. The data needed to reproduce S4 Fig can be found in S3 Data. (TIFF) [file pbio.3003081.s005.tif]

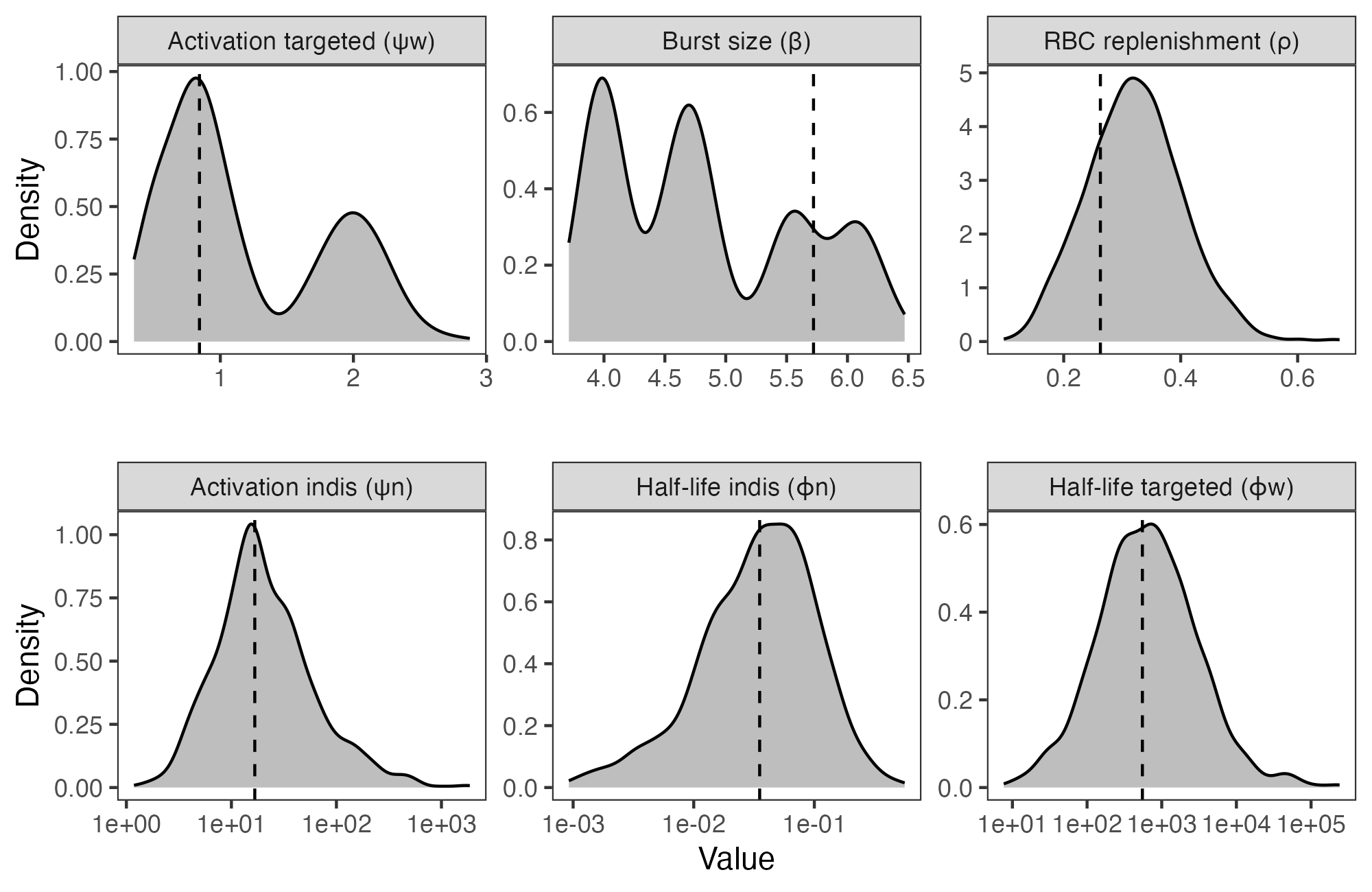

Supplement: S6 Fig — The histograms display the distribution of 1000 parameter values drawn from 167 Markov chains derived by Kamiya et al. [42]. The parameter values used in the deterministic model are indicated by the dotted line. The data needed to reproduce S6 Fig can be found in S9 Data. (TIFF) [file pbio.3003081.s006.tif]

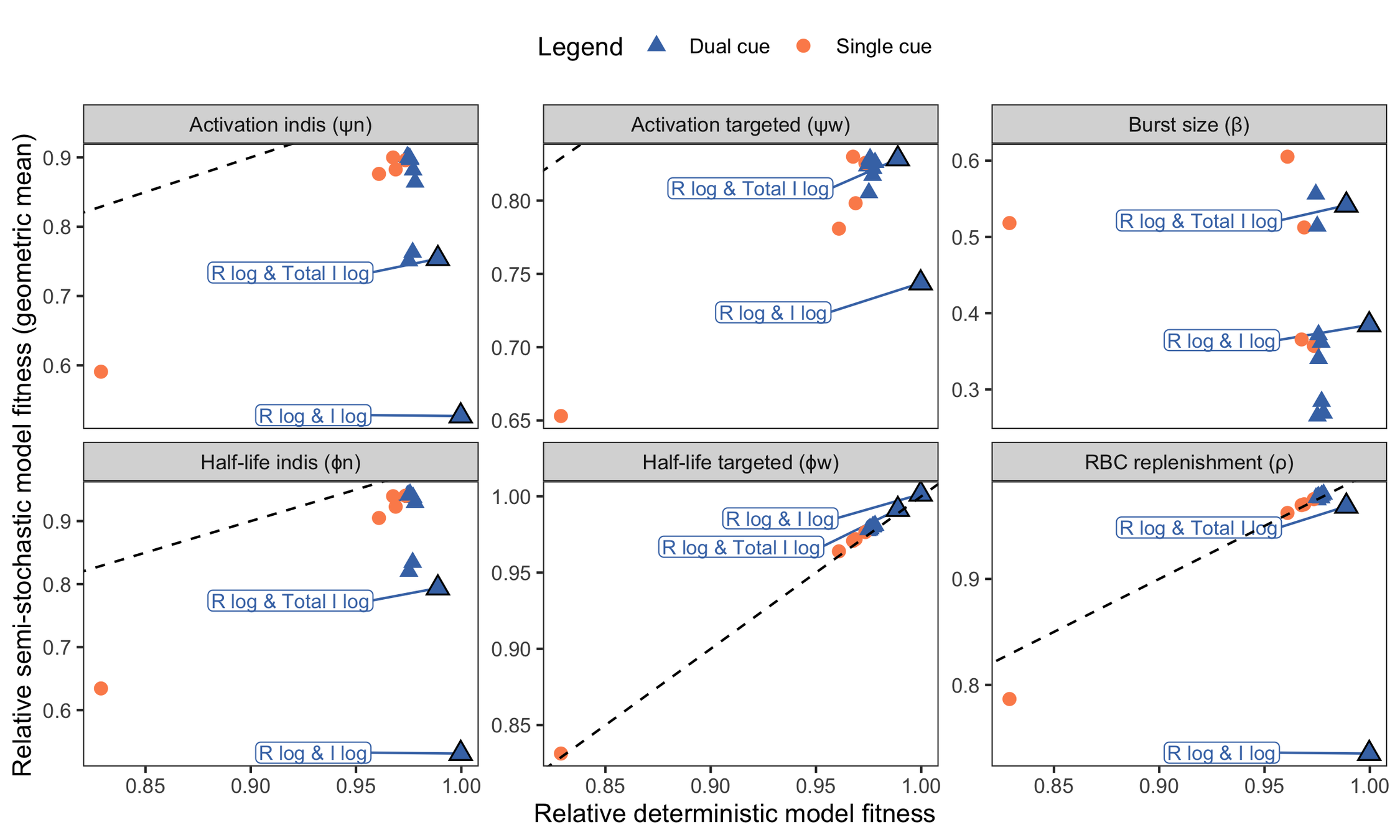

Supplement: S7 Fig — We conducted 1000 simulations, each lasting 20 days, in which parasites adopted the optimal transmission investment strategy (from the deterministic model) using a semi-stochastic model where only one parameter was randomly drawn from the posterior distributions (see S6 Fig). For each parameter that is varied (indicated in the facet panels), we plot the geometric mean of the fitness on the y-axis and the fitness from the deterministic model on the x-axis. All fitness values are normalized with respect to the best time-varying strategy. The dashed line represents a 1:1 relationship between the x-axis and the y-axis; the line is not visible on the plot for burst size, since variation in this parameter has the greatest negative influence on fitness. The data needed to reproduce S7 Fig can be found in S10 Data. (TIFF) [file pbio.3003081.s007.tif]
